# Supplementary material for: Joint inference of CFC lifetimes and banks suggests previously unidentified emissions
Source: Nat Commun. 2021 May 18;12:2920. doi: 10.1038/s41467-021-23229-2 (PMC8131697; doi:10.1038/s41467-021-23229-2)
Supplement: Supplementary file 1 — Supplementary Information [file 41467_2021_23229_MOESM1_ESM.pdf]

Joint inference of CFC lifetimes and banks suggests previously unidentified emissions

Supplementary Information

Megan Lickley[1*], Sarah Fletcher[2], Matt Rigby[3] and Susan Solomon[1]

1. Department of Earth, Atmospheric, and Planetary Sciences, Massachusetts Institute of Technology, Cambridge, MA 02139, USA.
2. Civil and Environmental Engineering, Stanford University, Stanford, CA, 94305, USA
3. School of Chemistry, University of Bristol, Bristol, BS8 1QU, UK

* corresponding author: mlickley@mit.edu

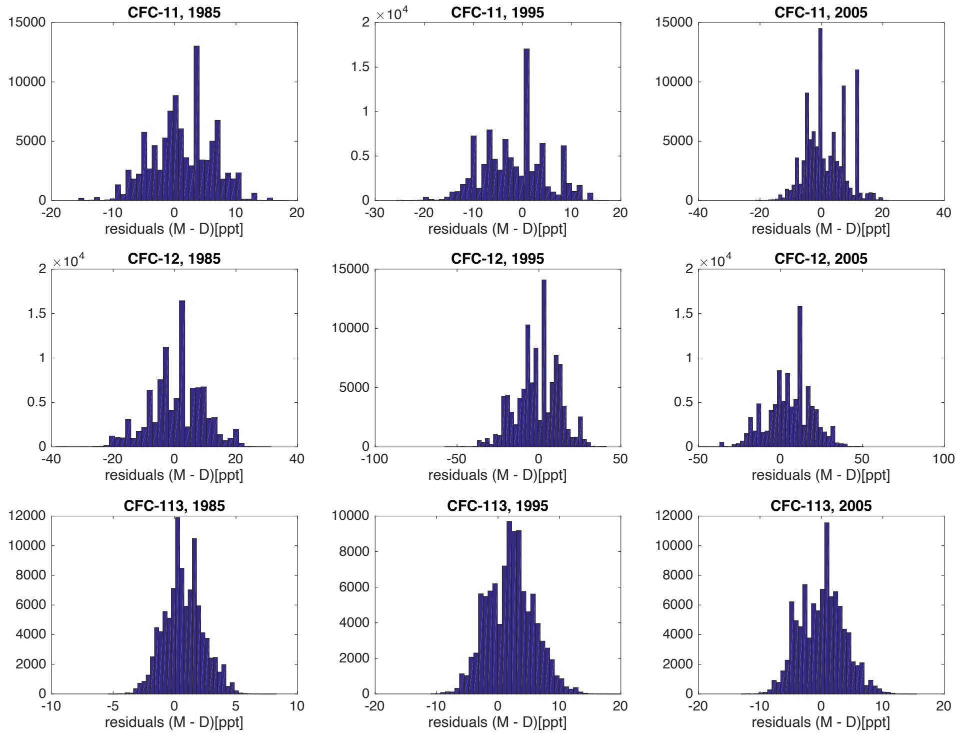

Supplementary Figure 1: Posterior residuals for each molecule for three representative years in the analysis (1985, 1995, and 2005).

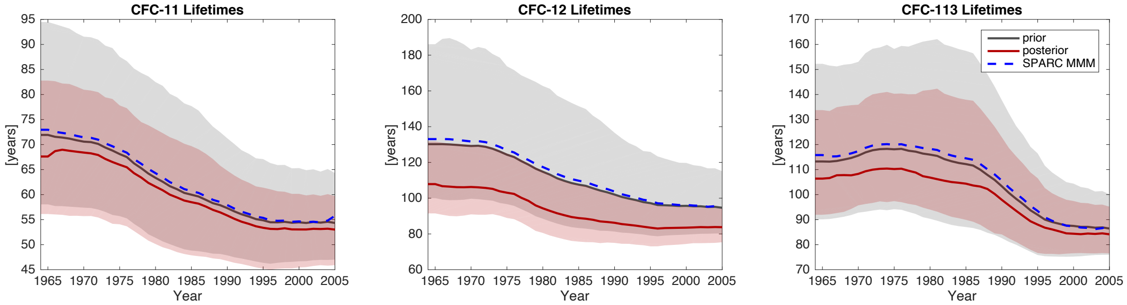

Supplementary Figure 2: Lifetime prior and posterior estimates for independent lifetime scenario.

Supplementary Table 1: Posterior lifetime estimates for the unexpected emissions scenario, without dependencies included (corresponding to Figure S2).

	**CFC-11**	**CFC-12**	**CFC-113**
**Median Posterior for 2010 (95% CI)**	53.0 (45.8, 59.6)	83.8 (75.3, 94.1)	84.2 (76.7, 95.3)
**Median time averaged lifetime (95% CI)**	60.1 (50.2, 70.2)	93.6 (81.3, 110.5)	99.4 (86.1, 120.2)

Supplementary Table 2: Posterior lifetime estimates for the reported emissions scenario, with dependencies included.

	**CFC-11**	**CFC-12**	**CFC-113**
**Median Posterior for 2010 (95% CI)**	49.5 (45.3, 55.3)	86.6 (77.1, 96.2)	80.3 (74.3, 88.0)
**Median time averaged lifetime (95% CI)**	55.1 (49.4, 63.6)	96.7 (83.5, 110.7)	92.1 (81.7, 104.9)

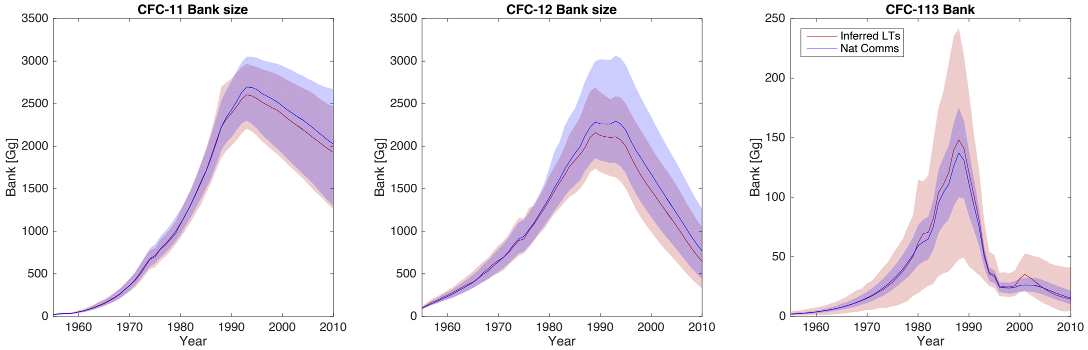

Supplementary Figure 3: Comparison between Lickley et al. (2020)[1] published bank estimates (blue) and new bank estimates (red). Uncertainties in CFC-113 banks are a reflection of wider production priors used in this analysis.

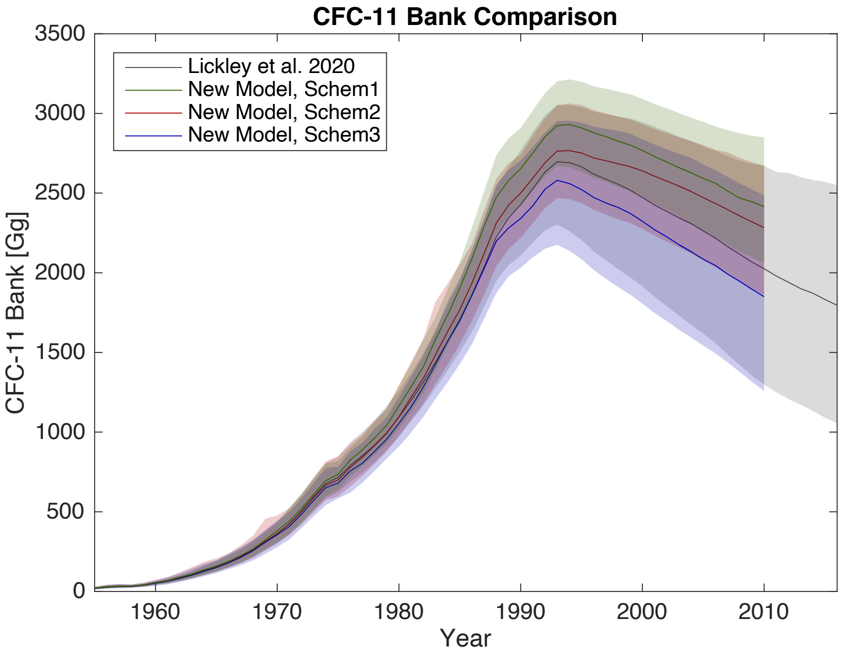

Supplementary Figure 4: Bank estimate comparisons across model configurations and to the Lickley et al. (2020)[1] bank estimate. This figure provides a comparison across model configurations. For each scenario, the lifetime is the SPARC MMM and the unexpected emissions scenario is assumed for a consistent comparison. The grey is the Lickley at al. bank estimate. Scheme 1 is the same as Lickley et al. but with the updated likelihood function. Scheme 2 is the same as Scheme 1 but uses the updated RF and DE functions. Scheme 3 is the new model configuration, which is the same as Scheme 2 but now infers atmospheric lifetimes.

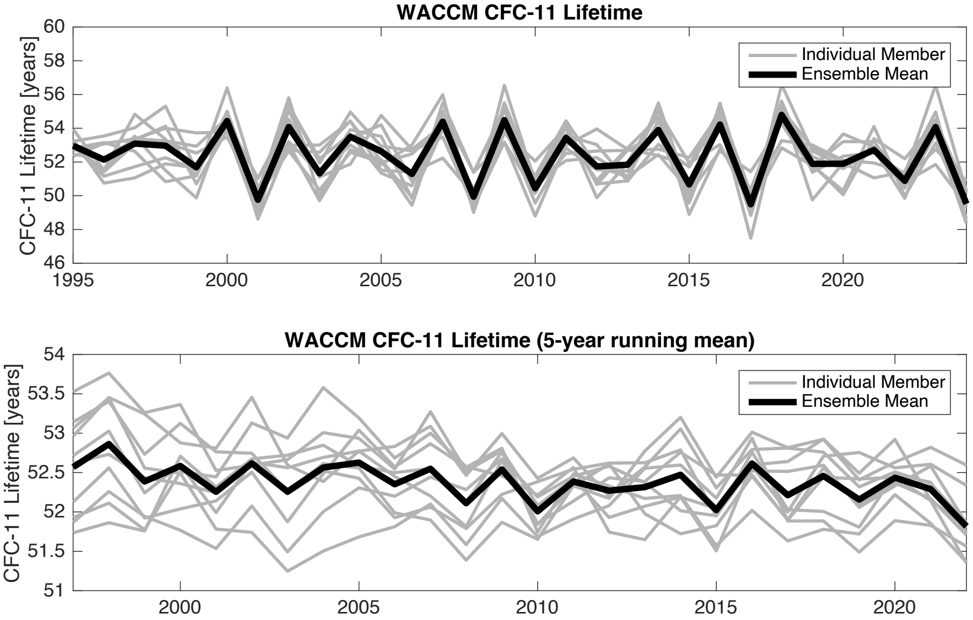

Supplementary Figure 5: Ensemble modeled lifetimes from the Whole Atmosphere Chemistry Climate Model (WACCM) including a repeating 28-month QBO forcing.

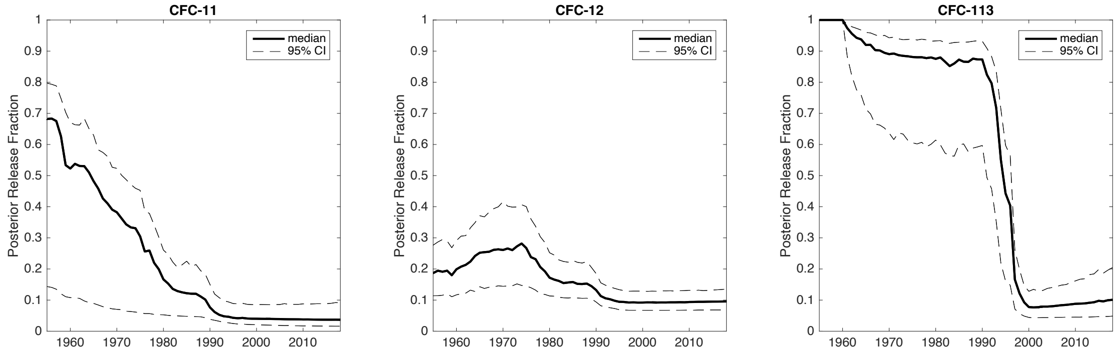

Supplementary Figure 6: Posterior release fraction for reported production scenario.

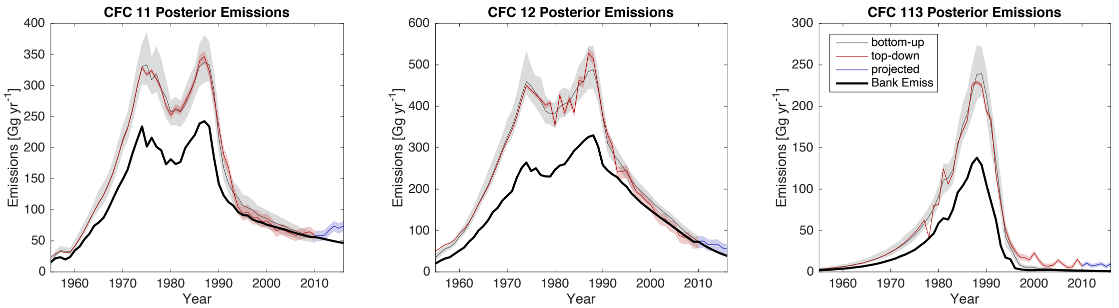

Supplementary Figure 7: Posterior emissions (red and grey), median posterior bank emissions (black), observationally derived emissions with posterior lifetimes (blue) for the reported production scenario

Supplementary Table 3: Average emissions (95% CI) by source for the reported production scenario. Values are reported in [Gg/yr].

	CFC-11	CFC-12	CFC-113
**Bank Emissions 2002-2012**	61.0<br>(50.3, 68.8)	92.0<br>(75.0, 104.9)	1.9<br>(1.2, 3.0)
**Total Emissions 2002-2012**	62.3<br>(53.2, 71.1)	93.2<br>(76.6, 105.2)	8.9<br>(6.1, 11.6)
**Direct Total Emissions 2002-2012**	1.1<br>(-7.0, 9.0)	0.6<br>(-17.8, 23.3)	7.0<br>(3.8, 9.4)
**Bank Emissions 2014-2016**	48.0<br>(41.7, 52.9)	43.1<br>(26.9, 57.8)	1.0<br>(0.5, 1.5)
**Total Emissions 2014-2016**	72.8<br>(64.3,81.0)	60.2<br>(44.2,71.9)	8.7<br>(6.1, 11.2)
**Direct Total Emissions 2014-2016**	24.9<br>(17.9, 31.0)	16.7<br>(-3.1, 37.9)	7.7<br>(5.0, 10.1)

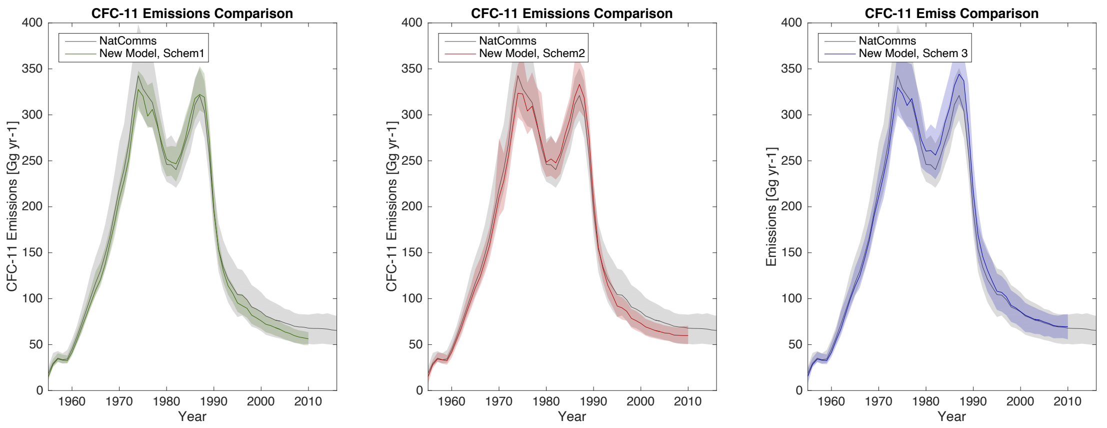

Supplementary Figure 8: Posterior bottom-up posterior emissions comparisons across model configurations and to the Lickley et al. (2020)[1] time series. All time series are for the fugitive emissions scenario. All panels show the Lickley et al. emissions in grey. Scheme 1, shown on the left, is the same as Lickley et al. but with the updated likelihood function. Scheme 2, shown in the middle, is the same as Scheme 1 but uses the updated RF and DE functions. Scheme 3, shown on the right, is the new model configuration, which is the same as Scheme 2 but also infers atmospheric lifetimes.

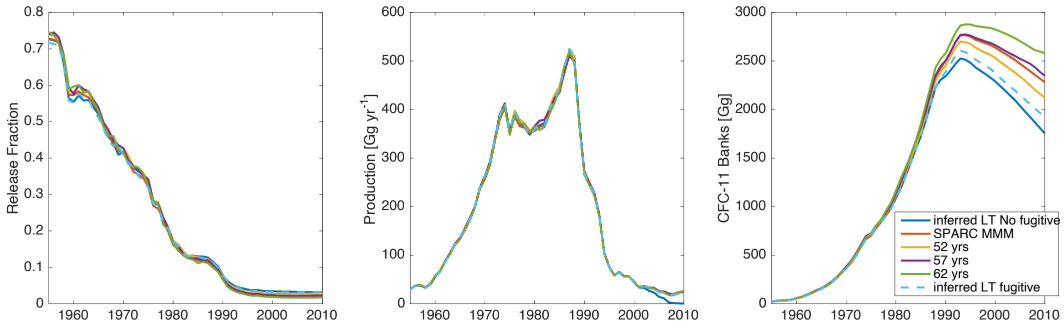

Supplementary Figure 9: Comparison of inferred median parameter values for different scenarios.

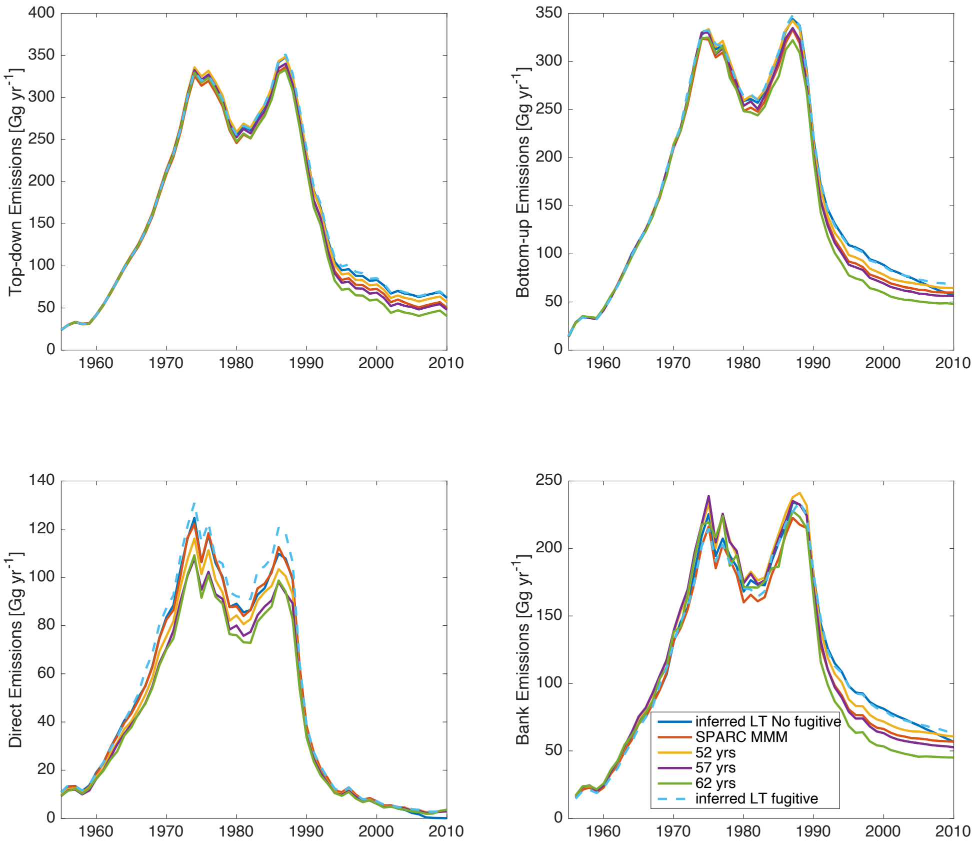

Supplementary Figure 10: Comparison of inferred median CFC-11 emissions values from BPE analysis using prescribed lifetime scenarios compared to inferred lifetimes. The top left panel shows the top-down (or observationally-derived) emissions for each scenario. The inferred LT scenarios are depicted by their median top-down inferred values. For all other lifetime scenarios, lifetime values are assumed and treated as known. The top-left panel shows the corresponding bottom-up median inferred emissions values. The bottom-left panel shows the direct total inferred emissions and the bottom-right panel shows inferred bank emissions for each scenario.

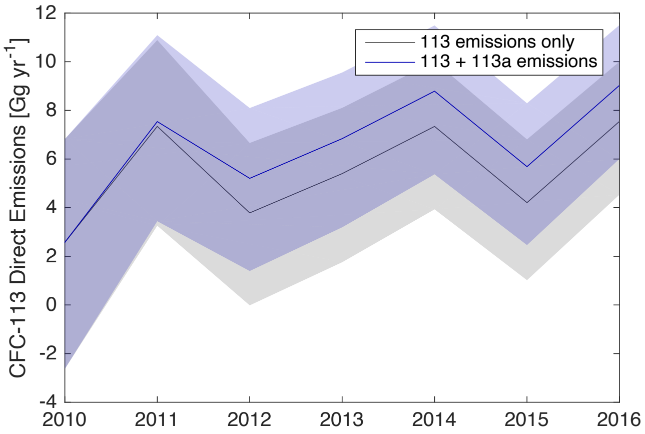

Supplementary Figure 11: Direct total emissions of CFC-113. The blue line shows the estimated emissions of CFC-113 using AGAGE NOAA dataset which does not separate CFC-113a from total CFC-113 measurements. The gray line shows the estimated emissions after removing the CFC-113a trend from Adcock et al. (2018)[2], assuming the instrument is equally sensitive to both CFC-113 and CFC-113a. Note that the BPE analysis does not separate the two isomers here. Instead, the emissions are estimated using the posterior 2010 lifetimes from the BPE analysis. Shaded region indicates the 95% confidence interval.

Supplementary References

1. Lickley, M. *et al*. banks to emissions and impacts on the ozone layer and climate. *Nat. Commun.* **11**, (2020).
2. Adcock, K. E. *et al*. Continued increase of CFC-113a (CCl3CF3) mixing ratios in the global atmosphere: Emissions, occurrence and potential sources. *Atmos. Chem. Phys.* **18**, 4737–4751 (2018).
